# PV and SST neurons in the anterior cingulate cortex regulate social disorders in adulthood induced by sensory abnormalities in childhood

**DOI:** 10.1111/cns.14863

**Published:** 2024-07-22

**Authors:** Xiang‐Dong Wan, Guo‐Hong Cai, Zi‐Qian Yan, Xue‐Qing Liu, Ding‐Ding Yang, Yi‐Fan Lu, Lei‐Ting An, Sheng‐Xi Wu, Fan Zhang

**Affiliations:** ^1^ The Key Laboratory of Neural and Vascular Biology, Ministry of Education and Department of Biochemistry and Molecular Biology Hebei Medical University Shijiazhuang China; ^2^ Department of Neurobiology, School of Basic Medicine Fourth Military Medical University Xi'an China; ^3^ Department of Nuclear Medicine Xijing Hospital, Fourth Military Medical University Xi'an China; ^4^ Graduate School of Hebei Medical University Shijiazhuang China

**Keywords:** anterior cingulate cortex, PV neurons, social disorder, SST neurons

## Abstract

**Objective:**

Childhood sensory abnormalities experience has a crucial influence on the structure and function of the adult brain. The underlying mechanism of neurological function induced by childhood sensory abnormalities experience is still unclear. Our study was to investigate whether the GABAergic neurons in the anterior cingulate cortex (ACC) regulate social disorders caused by childhood sensory abnormalities experience.

**Methods:**

We used two mouse models, complete Freund's adjuvant (CFA) injection mice and bilateral whisker trimming (BWT) mice in childhood. We applied immunofluorescence, chemogenetic and optogenetic to study the mechanism of parvalbumin (PV) neurons and somatostatin (SST) neurons in ACC in regulating social disorders induced by sensory abnormalities in childhood.

**Results:**

Inflammatory pain in childhood leads to social preference disorders, while BWT in childhood leads to social novelty disorders in adult mice. Inflammatory pain and BWT in childhood caused an increase in the number of PV and SST neurons, respectively, in adult mice ACC. Inhibiting PV neurons in ACC improved social preference disorders in adult mice that experienced inflammatory pain during childhood. Inhibiting SST neurons in ACC improved social novelty disorders in adult mice that experienced BWT in childhood.

**Conclusions:**

Our study reveals that PV and SST neurons of the ACC may play a critical role in regulating social disorders induced by sensory abnormalities in childhood.

## Graphical abstract

Inflammatory pain in childhood leads to social preference disorders, while BWT in childhood leads to social novelty disorders in adult mice. Inflammatory pain and BWT in childhood caused an increase in the number of PV and SST neurons, respectively, in adult mice ACC. Inhibiting PV neurons in ACC improved social preference disorders in adult mice that experienced inflammatory pain during childhood. Inhibiting SST neurons in ACC improved social novelty disorders in adult mice that experienced BWT in childhood. PV and SST neurons of the ACC may play a critical role in regulating social disorders induced by sensory abnormalities in childhood.

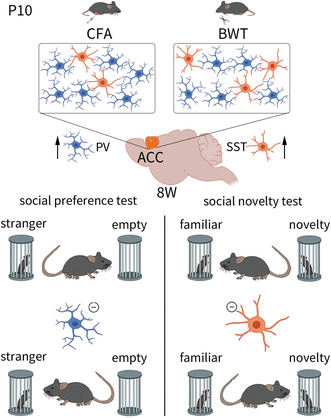

## 1 INTRODUCTION

In the early development process of newborns after birth, neural connections in the brain are established according to genetic programs, and the brain undergoes extensive processing to adapt to constantly changing environments.^1^, ^2^ If there are long‐term repeated adverse experiences in childhood that deviate from the expected growth environment, the brain makes more adjustments to adapt, which may lead to mental and physical health problems in adulthood.^3^ Numerous studies have reported that long‐term changes in the immune system and neurobiology during childhood may have a serious impact on perception and emotions in adulthood.^4^, ^5^, ^6^ Adverse childhood experiences can alter the brain at multiple levels, but the mechanisms are not yet fully understood.^7^, ^8^, ^9^

Social disorder is one of the abnormal states that can occur in adulthood after childhood negative experiences. Research has found that cutting off the beard of mice 12–16 days after birth can affect the formation of neural circuits related to the tactile system of the beard, leading to social disorders in mice as adults.^10^ Being socially isolated during childhood can also lead to social barriers in adulthood.^11^ Previous research found that chronic pain in adult mice could lead to social disorders in mice.^12^ This raises the question of whether chronic pain during childhood in mice would cause social disorders in adult mice.

Neuroimaging studies on adverse childhood experiences such as chronic pain in children have found significant changes in multiple brain regions in adulthood.^13^ Children with autism not only have social disorders, but also have sensory abnormalities, which have a significant impact on brain development.^14^, ^15^ The anterior cingulate cortex (ACC) plays a crucial role in pain,^16^ as well as in regulating social behavior.^17^, ^18^ Normal ACC function depends on the balance of neuronal excitation/inhibition. γ‐ Aminobutyric acid (GABA) is the main inhibitory neurotransmitter in the nervous system that plays an important role in regulating neuronal activity. Previous data suggests the regulatory role of GABAergic neurons in pain and social regulation in the ACC.^19^, ^20^, ^21^ Therefore, another line of query is whether GABAergic neurons in the ACC also play an important role in adverse experiences such as chronic pain and tactile abnormalities during childhood.

In this study, we clarified whether chronic pain during childhood in mice affected social behavior in adult mice, as well as the mechanism of action of GABAergic neurons in the ACC in adult social disorders caused by different types of adverse childhood experiences.

## 2 METHODS

### 2.1 Experimental animals

This study used 10 days old and 8 weeks old C57/BL6J mice provided by the Experimental Animal Center of the Fourth Military Medical University. PV‐Cre mice and SST‐Cre mice were purchased from Jiangsu Jinzhihe Biotechnology Co., Ltd. All programs have been approved by the Institutional Animal Care and Use Committee (FMMU) of the Fourth Military Medical University and comply with the guidelines for experimental animal care and use published by the National Institutes of Health (NIH) in the United States. The mice were placed in a constant temperature room, with a temperature of 22–24°C and an average humidity of 50%, and underwent a 12 h light/dark cycle (with light from 08:00 to 20:00). Water and food can be provided freely.

### 2.2 Animal models

Inflammatory pain model: 10 days after birth, 20 μL CFA was injected into the right hind paw of mice, while 20 μL saline was injected into the right hind paw of mice the control group. Bilateral Whisker Trimming (BWT) model: At 10 days or 8 weeks after birth, mice were given BWT, while the beard of the control group was not removed.

### 2.3 Three‐chamber test

The testing device consists of three chambers (40 × 20 cm^2^ each) and two binding cages (17 cm high). The entire experimental process is divided into three stages. In the first stage, the experimental animals are placed in the middle chamber for 5 min to adapt to the entire device; The second stage is a social preference test, in which an stranger mouse of the same gender is placed in a cage in the left chamber, and an empty cage is placed in the right chamber for 10 min; The third stage is a social novelty test, in which an novelty mouse of the same gender is placed in a cage in the right chamber and recorded for 10 min. Use SMART3.0 software to analyze the time spent by mice at different stages in each chamber.

### 2.4 Pain threshold test

To test the mechanical pain threshold, we placed the mice in a box on an elevated metal mesh, adapted for about 40 min, and then stimulated their right hind foot with a series of logarithmically increasing fibers perpendicular to the surface of the central sole, determining a 50% paw retraction threshold. Using the Hargreaves radiation thermal instrument for thermal pain threshold testing, the latent period of basal claw retraction was adjusted to 9–12 s with a cutoff time of 25 s to prevent tissue damage. Each mouse was tested three times with a 10 min interval between each test, and the average of the three tests was taken as the final thermal pain threshold result.

### 2.5 Virus injection and fiber optic implantation

Stereotactic surgery: Mice were anesthetized with 1% pentobarbital sodium (60 μL/g), and their heads were fixed on a stereotactic adapter. Microinjector and microinjection pump were used to inject 200 nL of virus into bilateral ACC at a speed of 35 nL/min. The three‐dimensional positioning coordinates for ACC injection are 0.8 mm on the anterior posterior side (AP), 0.3 mm on the medial and lateral side (ML), and 1.75 mm on the dorsal ventral side (DV). One week after injection of the optogenetic virus, a fiber optic plug is implanted at the injection site, with the coordinates of the plug about 200 microns above the virus injection coordinates. When ACC is implanted with fiber optic cables on both sides, calculate the fiber optic cable position at a 15 degree angle according to the formula, and place the rotating arm at an angle of 15 degrees at the origin of the anterior fontanelle, according to AP = 0.8 mm; ML = 0.74 mm; DV = 1.26 mm is the fiber optic implantation site after adjusting the rotating arm. Before conducting behavioral tests, the mice recovered from surgery for at least 2 weeks.

Using the following virus: 1. rAAV‐EF1a‐DIO‐mCherry (titer: 3.12 × 10^12^ vg/mL; BrainVTA) 2. rAAV‐EF1a‐DIO‐eNpHR3.0‐mCherry (titer: 2.18 × 10^12^ vg/mL; BrainVTA) 3. rAAV‐EF1a‐DIO‐mCherry (titer: 2.43 × 10^12^ vg/mL; BrainVTA) 4. rAAV EF1a‐DIO‐hM4D (Gi)‐mCherry (titer: 2.13× 10^12^ vg/mL; BrainVTA). All virus vectors were referenced and stored at −80°C until use.

### 2.6 Chemogenetics

After injection of chemical genetic virus mCherry or hM4D (Gi), the mice recovered for 4 weeks. After intraperitoneal injection of 0.1 mg/mL CNO (Clozapine N‐oxide) (1 mg/kg) into mice for 40 min, three‐chamber test began.

### 2.7 Optogenetics

After injection of mCherry or eNpHR virus, mice recovered for 4 weeks. Before conducting behavioral tests, the mice were placed in a behavioral room and acclimated for 50 min. Connect the jumper to the fiber optic cable on the mouse head and control the laser light source through a waveform generator. Set the 589 nm yellow light to continuous light with a power of 5–7 mW. Start behavioral testing after turning on the laser.

### 2.8 Immunofluorescence

Anesthetize mice with 1% pentobarbital sodium (60 μL/g), perfuse 25 mL of pre cooled 0.01 MPBS (pH = 7.4) through the heart, then reperfusion 50 mL of 4% paraformaldehyde, remove mouse brain tissue, fix with 4% PFA for 2 h, and dehydrate in a 30% sucrose solution for 36 h. Slice brain tissue using a cryoslicer, with each slice measuring 35 μm. Collect frozen coronal sections of ACC continuously in 0.01 M phosphate buffer. Free floating slices were pre incubated in a closed solution containing 3% normal bovine serum and 0.3% Triton X‐100 Tris buffer control (pH = 7.4), and left at room temperature for 1 h. Parvalbumin (PV) antibody (Swant, PV27‐200; μ l. 1:500), SST antibody (Sigma Aldrich, HPA019472, 1:400), and DAPI (Sigma Aldrich, D9564, 1:800) were dissolved in antibody diluent and incubated overnight with slices at 4°C. After washing three times, the slices were incubated with secondary antibody (Thermo Fisher) for 2 h, which was coupled with Alexa 488 or Alexa 594 at room temperature. The slices were observed under a confocal laser scanning microscope (Olympus FV3000).

### 2.9 Statistical analysis

All data were analyzed in GraphPad Prism 9.0 and SPSS, and presented as mean ± standard error (Mean ± SEM). The statistical methods used include unpaired *t*–test and Mann Whitney test. When *p* < 0.05, the difference is considered statistically significant.

## 3 RESULTS

### 3.1 Result 1: Inflammatory pain in childhood leads to social preference disorders in adult mice

**FIGURE 1:**
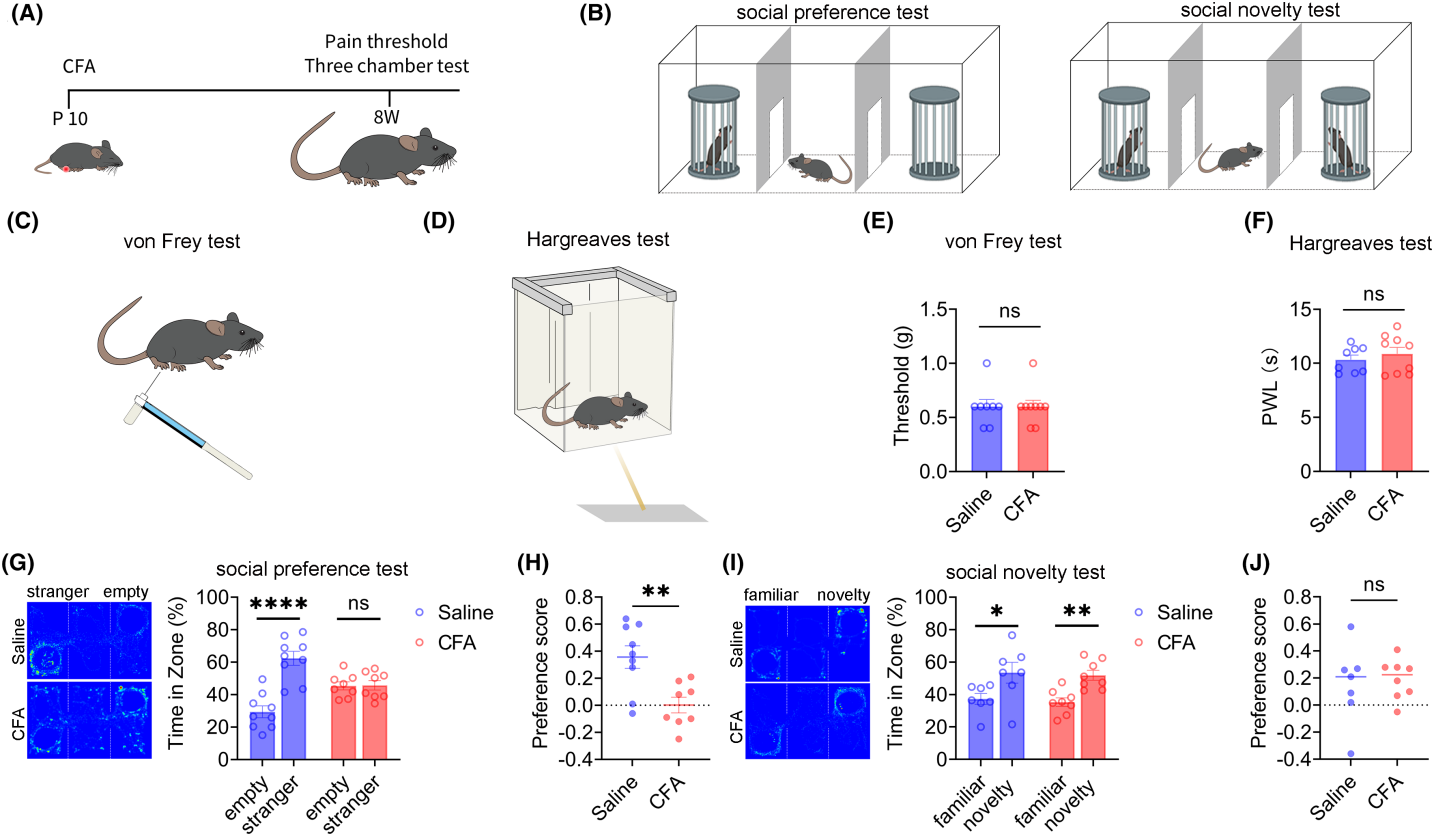
Inflammatory pain in young mice leads to social preference disorder in adults. (A) Experimental Flow Chart. (B–D) Experimental diagram shows von Frey test, Hargreaves test and three‐chamber test. (E) There was no difference in mechanical pain in adult mice between Saline and CFA groups (Saline: *N* = 8; CFA: *N* = 9, Mann Whitney test). (F) There was no difference in thermal pain in adult mice between Saline and CFA groups (Saline: *N* = 8; CFA: *N* = 9, Unpaired *t‐*test). (G) Saline group showed normal social preference, CFA group showed social preference disorder(Saline: *N* = 9, Unpaired *t*‐test; CFA: *N* = 8, Unpaired *t*‐test). (H) There was no difference in social index between Saline and CFA Group (Saline: *N* = 9; CFA: N = 8 Unpaired *t*–test).(I) Both Saline and CFA groups showed normal social novelty (Saline: *N* = 7, Unpaired *t*‐test; CFA: *N* = 8, Unpaired *t*‐test).(J) There was no difference in social index between Saline and CFA Group (Saline: *N* = 7; CFA: *N* = 8, Unpaved *t*‐test). All data are represented as mean ± standard error. See Supplementary Table for detailed statistical information. *p < 0.05, **p < 0.01, ***p < 0.001, ****p < 0.0001.

Injecting CFA into the right foot of mice on the 10th day of birth induced inflammatory pain models, with the control group injected with 20 μL saline. The flowchart of the experimental design is shown in Figure 1A. After 8 weeks, the pain threshold of mice was tested using the von Frey and Hargreaves tests (Figure 1C, D), and the social behavior of mice was tested using the three‐chamber test (Figure 1B). The pain threshold tests showed no significant difference in mechanical and thermal pain between the saline or CFA groups of adult mice (Figure 1E, F), indicating that inflammatory pain in mice injected with CFA 10 days after birth returned to normal in adulthood. Next, the three‐chamber test showed that during the social preference phase, the saline group mice spent significantly more time in the chamber with a stranger mouse than in an empty cage, indicating that these mice had normal social preferences. However, there was no significant difference in the time spent by the CFA group mice in the chamber with a stranger mouse compared with the time spent in the empty cage, indicating that the CFA group mice exhibited social preference disorders (Figure 1G). The social preference index of the CFA group mice was also significantly lower than that of the saline group mice (Figure 1H). During the social novelty phase, both the saline and CFA groups of mice spent significantly more time in the chamber with a novelty mouse than a familiar mouse (Figure 1I), indicating that the social novelty phase of the saline and CFA groups was normal, and there was no significant difference in the social preference index between the two groups (Figure 1J). These results indicate that inflammatory pain in childhood leads to social preference disorders in adult mice.

### 3.2 Result 2: BWT in childhood leads to social novelty disorders in adult mice

**FIGURE 2:**
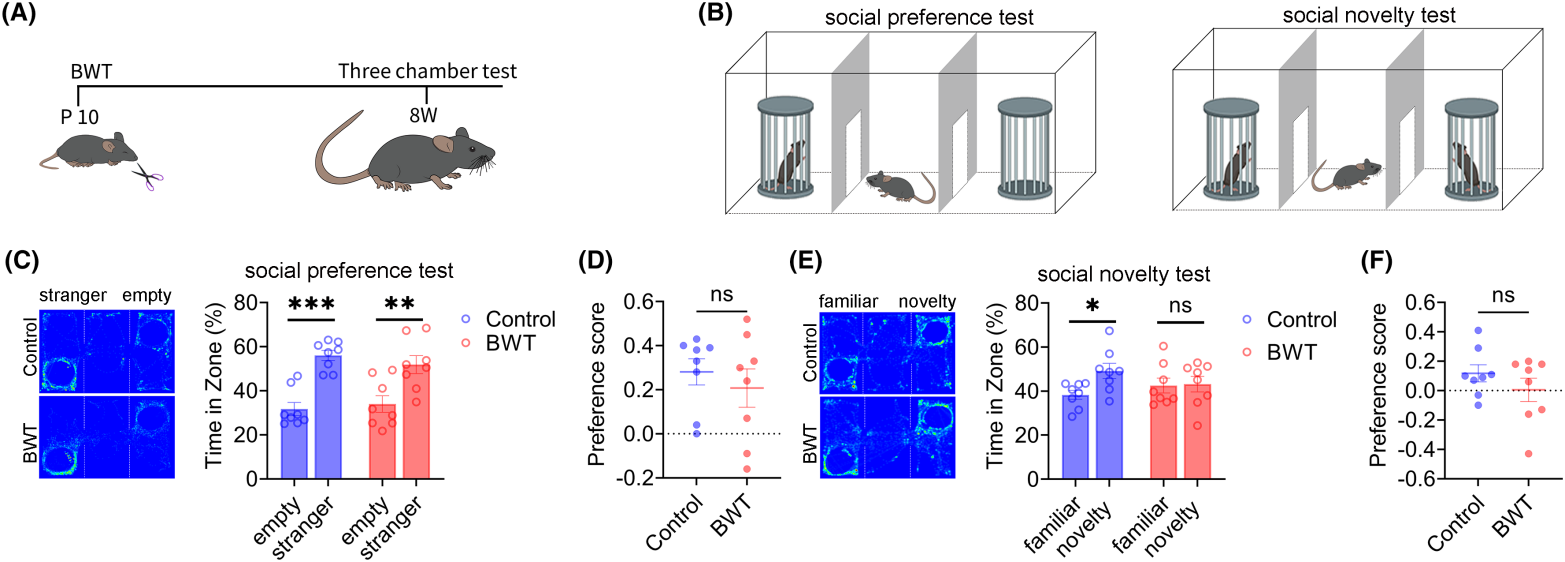
Bilateral whisker trimming (BWT) in young mice leads to social novelty disorder in adults. (A) Experimental Flow Chart. (B) Experimental diagram shows three‐chamber test. (C) Mice in Control and BWT Group showed normal social preference (Control: *N* = 7, Mann Whitney test; BWT: *N* = 8, Unpaired *t*‐test). (D) There was no difference in social index between control group and BWT group (control: *N* = 8, Mann Whitney test; BWT: *N* = 8, Mann Whitney test). (E) Control group mice showed normal social novelty. BWT group mice showed social novelty disorder (control: *N* = 8, Unpaired *t*‐test; BWT: *N* = 8, Unpaired *t*‐test). (F) There was no difference in social index between control group and BWT group (control: *N* = 8; BWT: *N* = 8, Unpaired *t*‐test). All data are represented as mean ± standard error. See Supplementary Table for detailed statistical information. *p < 0.05, **p < 0.01, *** *p* < 0.001, **** *p* < 0.0001.

Subsequently, we investigated the effect of BWT in childhood on the social behaviors of mice in adulthood. The flowchart of the experimental design is shown in Figure 2A. On the tenth day after birth, bilateral whiskers were removed from mice. At 8‐weeks old, the bilateral whiskers of mice had fully returned to normal (Figure 2A), and the social behaviors of the mice were tested (Figure 2B). The results of three‐chamber test showed that during the social preference stage, the control and BWT groups of mice spent significantly more time in the chamber with a stranger mouse than in the empty cage (Figure 2C), indicating that the social preference stage of the control and BWT groups of mice was normal, and there was no significant difference in the social preference index between the two groups (Figure 2D). In the social novelty stage, the control group mice spent significantly more time in the chamber with a novelty mouse than familiar mouse, while the BWT group mice spent a similar amount of time in the chamber with a novelty mouse and familiar mouse (Figure 2E), indicating that the BWT group mice had social novelty disorders, but there was no significant difference in the social preference index between the two groups (Figure 2F). The above results indicate that BWT in childhood leads to social novelty disorders in adult mice.

### 3.3 Result 3: BWT in adult mice cause social novelty disorde

**FIGURE 3:**
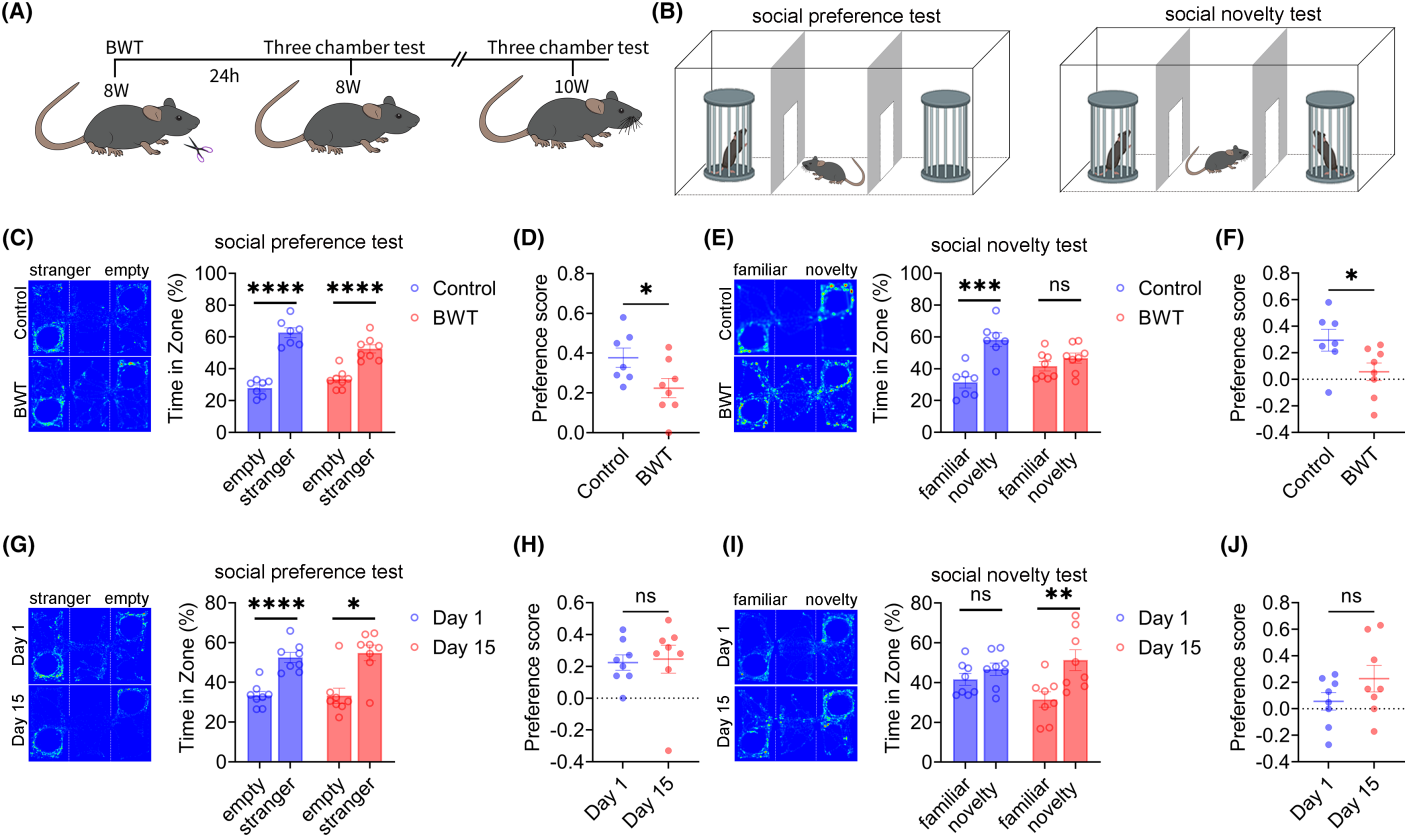
Bilateral whisker trimming (BWT) in adult mice leads to social novelty disorder. (A) Experimental Flow Chart. (B) Experimental diagram shows three‐chamber test. (C) Mice in Control and BWT Group showed normal social preference (Control: *N* = 7, Unpaired t test; BWT: *N* = 8, Unpaired *t*‐test). (D) The social index of control group was significantly higher than that of BWT group (control: *N* = 7; BWT: *N* = 8, Unpaired *t*‐test). (E) Control group mice showed normal social novelty. BWT group mice showed social novelty disorder (control: *N* = 7, Unpaired t test; BWT: *N* = 8, Unpaired *t*‐test). (F) The social index of control group was significantly higher than that of BWT group (control: *N* = 7; BWT: *N* = 8 Unpaired *t*‐test). (G) On the day1 and day15 after BWT, the mice showed normal social preference (Day1: *N* = 8, Unpaired t test; Day15: *N* = 8, Mann Whitney test). (H) There was no difference in social index between day1 and day15 after BWT (Day1: *N* = 8, Mann Whitney test; Day15: *N* = 8, Mann Whitney test). (I) On the day1 after BWT, the mice showed social novelty disorder; On the day15 after BWT, the mice showed normal social novelty (Day1: *N* = 8, Unpaired *t*–test; Day15: *N* = 8, Unpaired *t*‐test). (J) There was no difference in social index between day1 and day15 after BWT (Day1: *N* = 8, Unpaired *t*‐test; Day15: *N* = 8, Unpaired *t‐*test). All data are represented as mean ± standard error. See Supplementary Table for detailed statistical information. * *p* < 0.05, **p < 0.01, ***p < 0.001, ****p < 0.0001.

We also investigated the effects of BWT on social behaviors in adult mice. The flowchart of the experimental design is shown in Figure 3A. On the first day, bilateral whiskers were removed from adult mice, and on the second day, the social behaviors of the mice were tested (Figure 3B). The three‐chamber test results showed that both the control group and BWT group mice had normal social preference stages (Figure 3C), but the social preference index of the BWT group mice was lower than that of the control group (Figure 3D). In the social novelty stage, the control group was normal, while the BWT group showed social novelty disorders (Figure 3E), and the social preference index of the BWT group was lower than that of the control group (Figure 3F). The results indicate that BWT in adult mice also leads to social novelty disorders.

Next, we wanted to observe the social behaviors of adult mice when their whiskers returned to normal. After 15 days of BWT, adult mice's whiskers had returned to their normal length. The results of the three‐chamber test showed that on the 1st and 15th day after BWT, the social preference stage of the mice was normal (Figure 3G), and there was no significant difference in the social preference index between the two groups (Figure 3H). The social novelty stage, compared to the 1st day after BWT, returned to normal on the 15th day (Figure 3I), but there was no significant difference in the social preference index between the two groups (Figure 3J). The results suggest that social disorders induced by BWT in adulthood can return to normal when the whiskers of mice recover.

### 3.4 Result 4: Inflammatory pain and BWT in childhood cause an increase in the number of PV and SST neurons, respectively, in adult mice ACC

**FIGURE 4:**
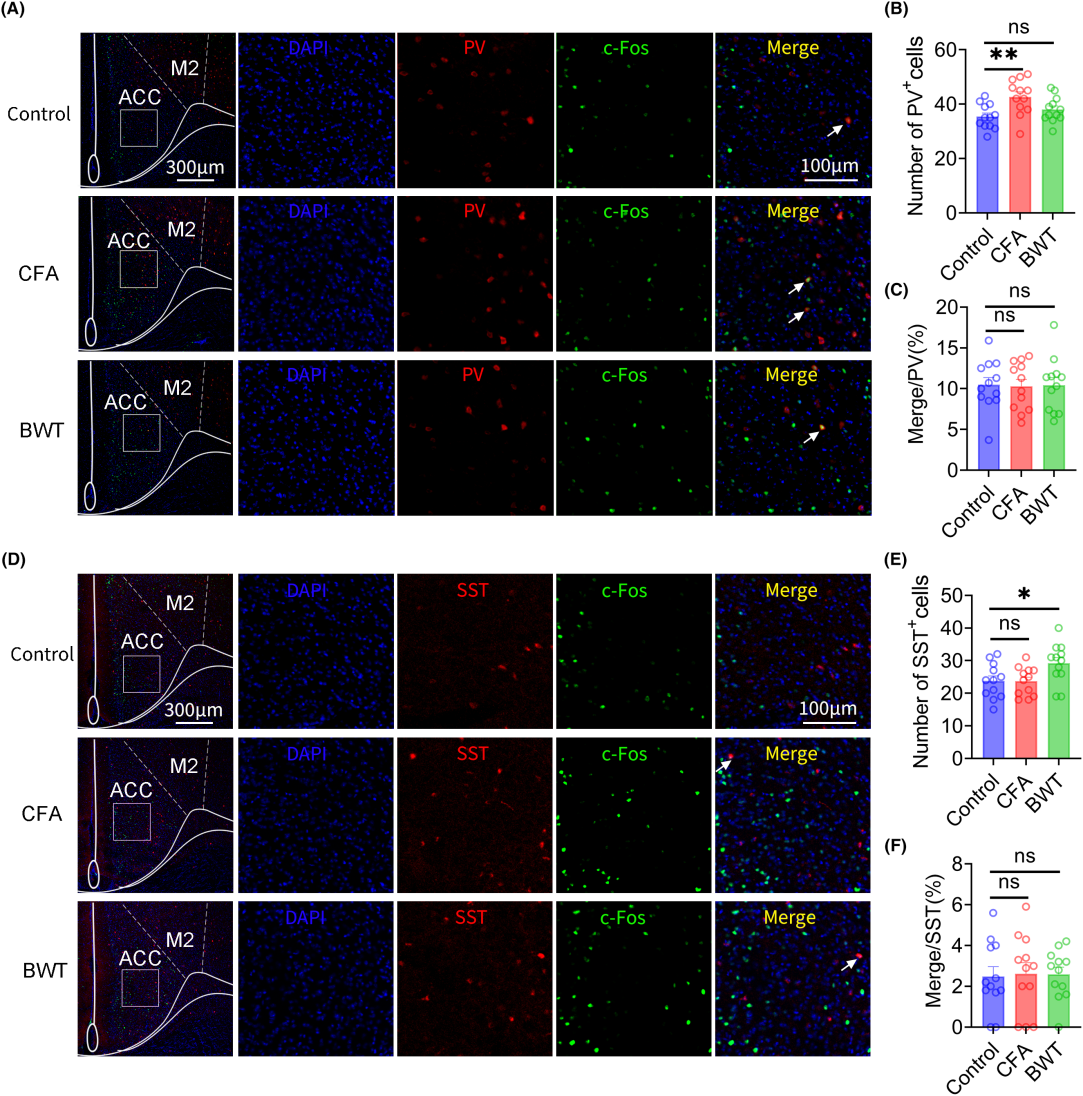
The number of ACC PV neurons increased in adult mice with juvenile inflammatory pain, and the number of ACC SST neurons increased in adult mice with bilateral whisker trimming. (A–C) Representative immunofluorescence images and quantitative analysis results of PV‐positive and c‐Fos positive neurons in ACC of mice in Control group, CFA group and BWT group (*n* = 12 from four mice in each group, Unpaired t test). (D–F) Representative immunofluorescence images and quantitative analysis results of SST‐positive and c‐Fos positive neurons in ACC of mice in Control group, CFA group and BWT group (*n* = 12 from four mice in each group, Unpaired *t*‐test). (Full scale: 1: 400 μm; Partial scale: 1:200 μm). All data are represented as mean ± standard error. See Supplementary Table for detailed statistical information. *p < 0.05, **p < 0.01.

Immunofluorescence staining was used to label PV and SST neurons in the ACC of adult mice that experienced inflammatory pain or BWT during childhood. We found that, compared with the control group, the number of PV neurons in the ACC increased in mice that experienced inflammatory pain in childhood. However, there was no significant difference in the number of PV neurons in mice that experienced BWT in childhood compared to that in the control group (Figure 4A, B). In addition, compared with the control group, there was no significant difference in the proportion of PV neurons and c‐Fos colocalization in ACC of adult mice that experienced inflammatory pain or BWT during childhood (Figure 4C). Furthermore, we found that, compared with the control group, the number of SST neurons in the ACC significantly increased in adult mice that experienced BWT in childhood. However, there was no significant difference in the number of SST neurons in mice that experienced inflammatory pain in childhood compared with that in the control group (Figure 4D, E). In addition, compared with the control group, there was no significant difference in the proportion of SST neurons and c‐Fos colocalization in ACC of adult mice that experienced inflammatory pain or BWT during childhood (Figure 4F).These results indicate that the social preference disorders induced by inflammatory pain in childhood may be related to an increase in the number of PV neurons and the social novelty disorders induced by BWT in childhood may be related to an increase in the number of SST neurons in the ACC in adulthood.

### 3.5 Result 5: Inhibiting ACC PV neurons can improve social preference disorders in adult mice that experienced inflammatory pain during childhood

**FIGURE 5:**
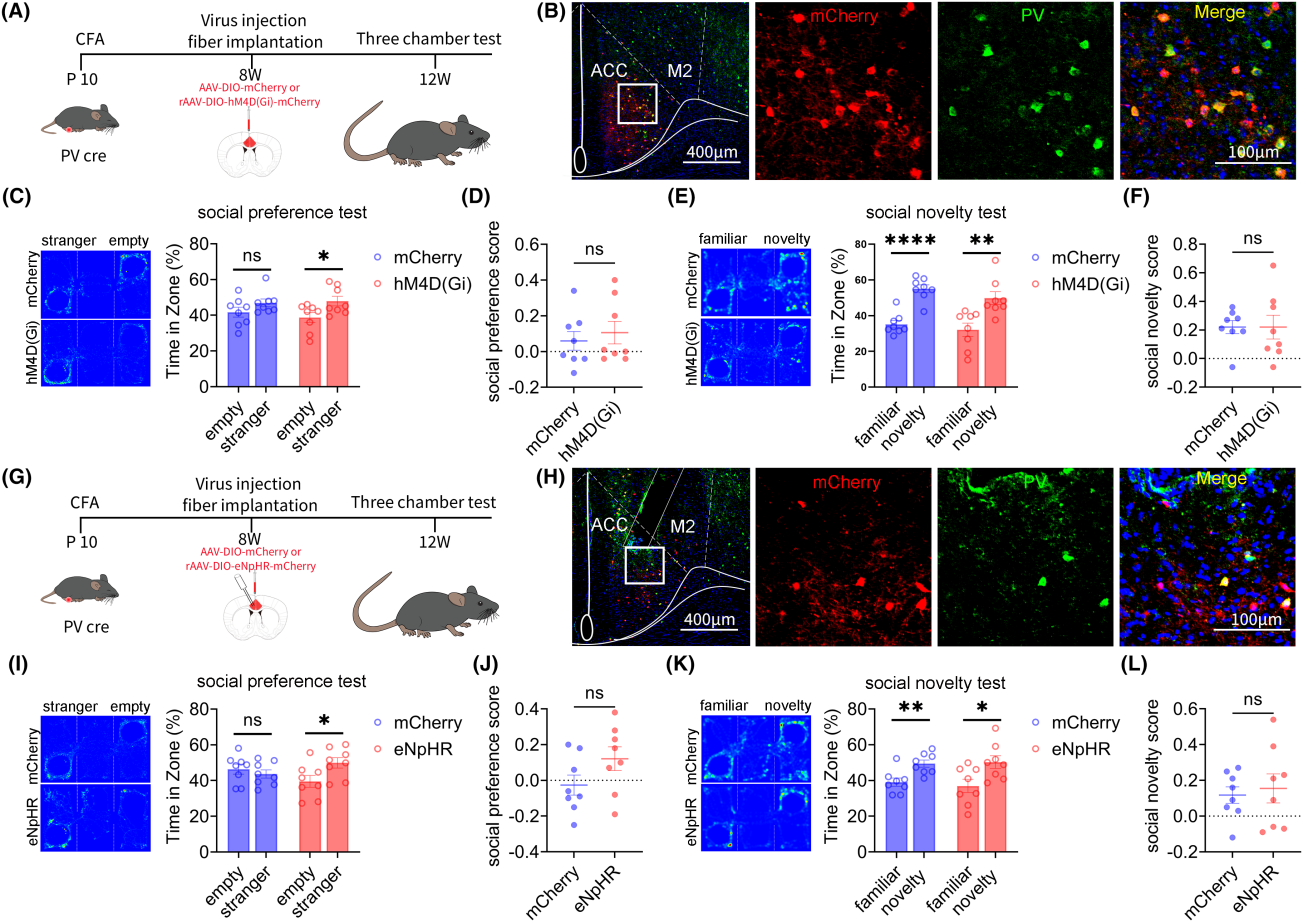
Inhibition of ACC PV neurons can improve the social preference disorder of young CFA model mice. (A) Chemogenetics experiment flow chart. (B) Representative images of mCherry (red) and PV interneurons (green) in the ACC (Full scale: 1: 400 μm; Partial scale: 1: 100 μm). (C) The mCherry group showed social preference disorder, while the hM4D (Gi) group showed normal social preference (mCherry: *N* = 6, Mann Whitney test; hM4D (Gi): *N* = 8, Unpaired t test). (D) There was no significant difference in social index between mCherry group and the hM4D (Gi) group (mCherry: *N* = 8; hM4D (Gi): *N* = 8, Mann Whitney test). (E) Mice in mCherry and hM4D (Gi) groups showed normal social novelty (mCherry: *N* = 8, Unpaired *t*‐test; hM4D (Gi): *N* = 8, Unpaired *t*‐test). (F) There was no significant difference in social index between the mCherry and the hM4D (Gi) group (mCherry: *N* = 8; hM4D (Gi): *N* = 8, Unpaired *t*‐test). (G) Optogenetics experiment flow chart. (H) Representative image of mCherry (red) combined with PV interneurons (green) in the ACC (Full scale: 1: 400 μm; Partial scale: 1: 100 μm). (I) The mCherry group showed social preference disorder, while the eNpHR group showed normal social preference (mCherry: *N* = 8, Unpaired *t*‐test; eNpHR: *N* = 8, Unpaired *t*‐test). (J) There was no significant difference in social index between the mCherry group and the eNpHR group (mCherry: *N* = 8; eNpHR: *N* = 8, Unpaired *t*–test). (K) Mice in mCherry and eNpHR groups showed normal social novelty (mCherry: *N* = 8, Unpaired *t*‐test; eNpHR: *N* = 8, Unpaired *t*‐test). (L) There was no significant difference in social index between the mCherry and the eNpHR group (mCherry: *N* = 8; eNpHR: *N* = 8, Unpaired *t*‐test). All data are represented as mean ± standard error. See Supplementary Table for detailed statistical information. * *p* < 0.05, **p < 0.01, ***p < 0.001, **** *p* < 0.0001.

Our previous research has shown that PV and SST neurons in the ACC regulate the social preference and social novelty stages, respectively. We further explored the role of PV neurons in the ACC social preference disorders induced by inflammatory pain in childhood, using chemogenetic and optogenetic methods. The flowchart of experimental design is shown in Figure 5A. On the 10th day after the birth of PV‐Cre mice, 20 μL CFA injections were administered to the feet of the mice. After 8 weeks, rAAV‐EF1a‐DIO‐mCherry or rAAV‐EF1a‐DIO‐hM4D (Gi)‐mCherry virus was injected into the bilateral ACC of mice. After 4 weeks of virus expression, the specificity of the virus was tested (Figure 5B). The three‐chamber tests showed that chemogenetic inhibition of ACC PV neurons improved social preference disorders induced by inflammatory pain in childhood, without affecting the social novelty stage of mice (Figure 5C–F).

Subsequently, the above results were further validated through optogenetics. The experimental design flow chart is shown in Figure 5G. CFA injections were administered to the feet of PV‐Cre mice on the 10th day after the birth. After 8 weeks, rAAV‐EF1a‐DIO‐mCherry or rAAV‐EF1a‐DIO‐eNpHR‐mCherry virus was injected into the bilateral ACC of the mice. After 4 weeks of virus expression, the specificity of the virus was tested (Figure 5H). The three‐chamber tests showed that Optogenetic inhibition of ACC PV neurons improved social preference disorders induced by inflammatory pain in childhood, without affecting the social novelty stage of mice (Figure 5I–L).

### 3.6 Result 6: Inhibiting ACC SST neurons can improve social novelty disorders in adult mice that experienced BWT in childhood

**FIGURE 6:**
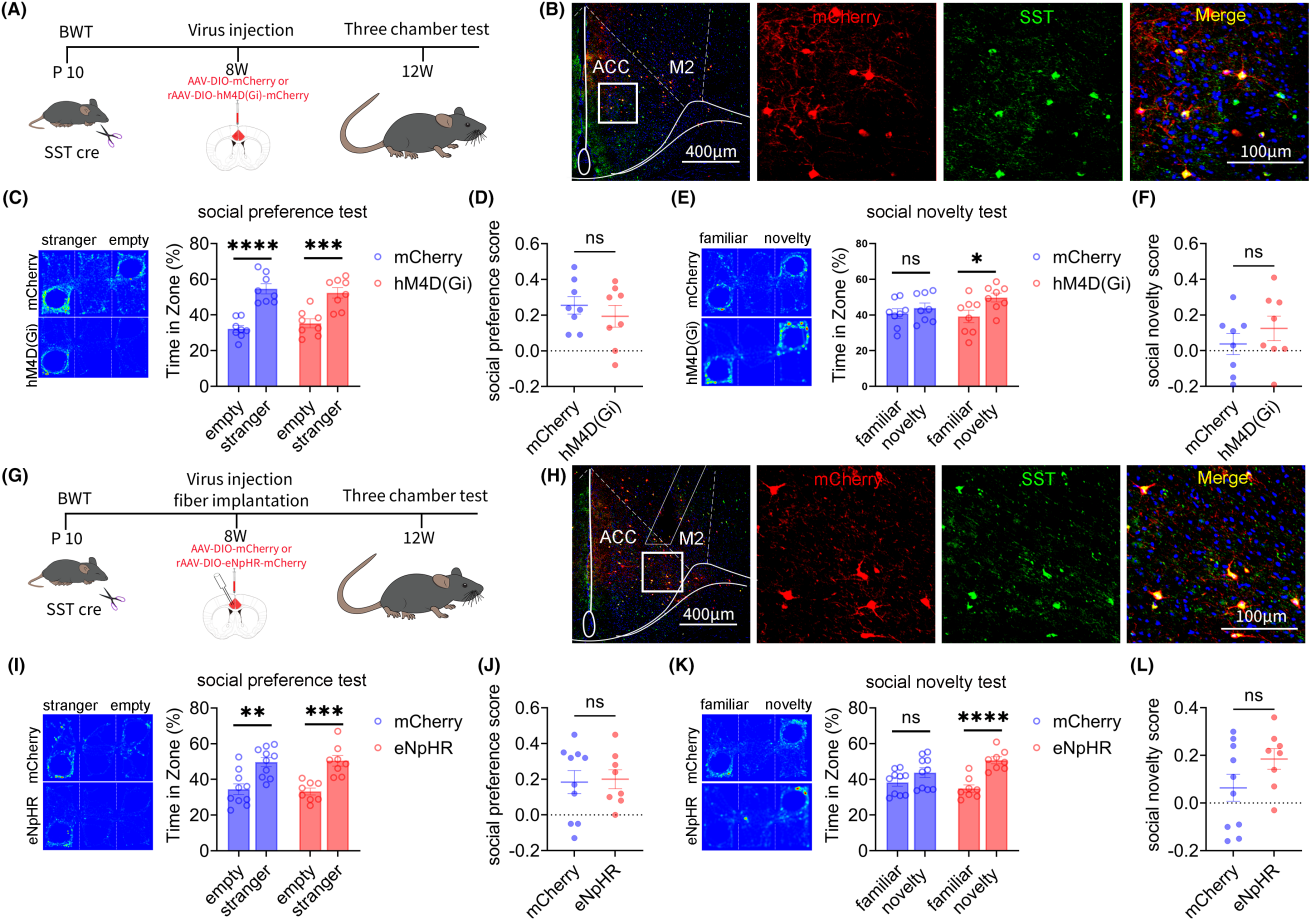
Inhibition of ACC SST neurons can improve the social novelty disorder of young BWT model mice. (A)Chemogenetics experiment flow chart. (B) Representative images of mCherry (red) combined with SST interneurons (green) in the ACC (Full scale: 1: 400 μm; Partial scale: 1: 100 μm). (C) Mice in mCherry and hM4D (Gi) groups showed normal social preference (mCherry: *N* = 8, Unpaired *t*‐test; eNpHR: *N* = 8, Unpaired *t*‐test). (D) There was no significant difference in social index between the mCherry and the hM4D (Gi) group (mCherry: *N* = 8; hM4D (Gi): *N* = 8, Unpaired *t*‐test). (E) The mCherry group showed social novelty disorder, while the hM4D (Gi) group showed normal social novelty (mCherry: *N* = 8, Unpaired t test; hM4D (Gi): *N* = 8, Unpaired *t*‐test). (F) There was no significant difference in social index between the mCherry and the hM4D (Gi) group (mCherry: *N* = 8; hM4D (Gi): *N* = 8, Unpaired *t*‐test). (G) Optogenetics experiment flow chart. (H)Representative image of mCherry (red) combined with SST interneurons (green) in the ACC (Full scale: 1: 400 μm; Partial scale: 1: 100 μm). (I) Mice in mCherry and eNpHR groups showed normal social preference. (mCherry: *N* = 10, Unpaired *t*‐test; eNpHR: *N* = 8, Unpaired *t*‐test). (J) There was no significant difference in social index between the mCherry and the eNpHR group (mCherry: *N* = 10; eNpHR: *N* = 8, Unpaired *t‐*test). (K) The mCherry group showed social novelty disorder, while the eNpHR group showed normal social novelty (mCherry: N = 10, Unpaired t test; eNpHR: *N* = 8, Unpaired *t*‐test). (L) There was no significant difference in social index between the mCherry and the eNpHR group (mCherry: *N* = 10; eNpHR: *N* = 8, Unpaired *t*‐test). All data are represented as mean ± standard error. See Supplementary Table for detailed statistical information. *p < 0.05, **p < 0.01, ***p < 0.001, ****p < 0.0001.

Next, we explored the role of ACC SST neurons in social novelty disorders in adult mice that experienced BWT in childhood, using chemogenetic and optogenetic methods. The flowchart of experiment design is shown in Figure 6A. On the 10th day after birth, the bilateral whiskers of SST‐Cre mice were removed. After 8 weeks, rAAV‐EF1a‐DIO‐mCherry or rAAV‐EF1a‐DIO‐hM4D (Gi)‐mCherry virus was injected into the ACC on both sides of the mice. After 4 weeks of virus expression, the specificity of the virus was tested (Figure 6B). The three‐chamber test showed that chemogenetic inhibition of ACC SST neurons improved social novelty disorders induced by BWT, without affecting their social preference stage (Figure 6C–F).

Subsequently, the above results were further validated through optogenetic methods. The experimental design flow chart is shown in Figure 6G. On the 10th day after birth, we removed the whiskers on both sides of SST‐Cre mice. After 8 weeks, we injected the rAAV‐EF1a‐DIO‐mCherry or rAAV‐EF1a‐DIO‐eNpHR‐mCherry virus into the ACC of the mice. After 4 weeks of virus expression, the specificity of the virus was tested (Figure 6H). The three‐chamber test showed that optogenetic inhibition of ACC SST neurons improved social novelty disorders induced by BWT, without affecting their social preference stage (Figure 6I–L).

### 3.7 Result 7: Inhibiting ACC SST neurons can improve social novelty disorders induced by BWT in adulthood

**FIGURE 7:**
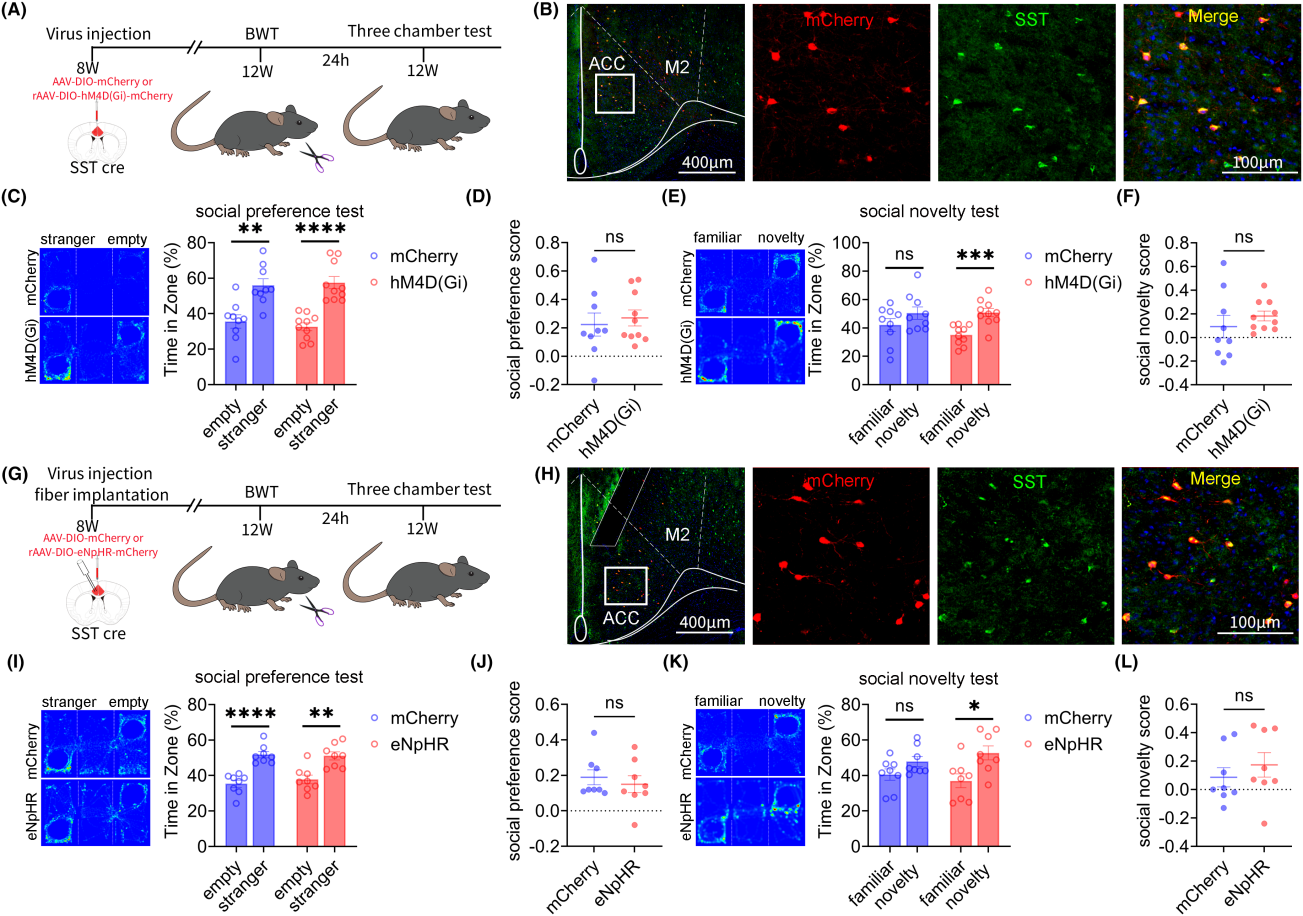
Inhibition of ACC SST neurons improves social novelty disorder in BWT model mice. (A) Chemogenetics experiment flow chart. (B) Representative image of mCherry (red) combined with SST interneurons (green) in the ACC (Full scale: 1: 400 μm; Partial scale: 1: 100 μm). (C) Mice in mCherry and hM4D (Gi) groups showed normal social preference (mCherry: *N* = 9, Unpaired *t*‐test; hM4D (Gi): *N* = 10, Unpaired *t*‐test). (D) There was no significant difference in social index between the mCherry and the hM4D (Gi) group (mCherry: *N* = 9; hM4D (Gi): *N* = 10, Unpaired *t*‐test). (E) The mCherry group showed social novelty disorder, while the hM4D (Gi) group showed normal social novelty (mCherry: *N* = 9, Unpaired t test; hM4D (Gi): *N* = 10, Unpaired *t*‐test). (F) There was no significant difference in social index between the mCherry and the hM4D (Gi) group (mCherry: *N* = 9; hM4D (Gi): *N* = 10, Unpaired *t*‐test). (G) Optogenetics experiment flow chart. (H) Representative image of mCherry (red) combined with SST interneurons (green) in the ACC (Full scale: 1: 400 μm; Partial scale: 1: 100 μm). (I) Mice in mCherry and eNpHR groups showed normal social preference. (mCherry: *N* = 8, Unpaired *t*‐test; eNpHR: *N* = 8, Unpaired *t*–test). (J) There was no significant difference in social index between mCherry and eNpHR group (mCherry: *N* = 8; eNpHR: *N* = 8, Mann Whitney test). (K) The mCherry group showed social novelty disorder, while the eNpHR group showed normal social novelty (mCherry: *N* = 8, Mann Whitney test; eNpHR: *N* = 8, Unpaired *t*‐test). (L) There was no significant difference in social index between the mCherry and the eNpHR group (mCherry: *N* = 8; eNpHR: *N* = 8, Unpaired *t*‐test). All data are represented as mean ± standard error. See Supplementary Table for detailed statistical information. *p < 0.05, **p < 0.01, ***p < 0.001, ****p < 0.0001.

We also investigated the role of ACC SST neurons in social novelty disorders induced by BWT in adulthood. The flowchart of experiment design is shown in Figure 7A. rAAV‐EF1a‐DIO‐mCherry or rAAV‐EF1a‐DIO‐hM4D (Gi)‐mCherry virus was injected into the bilateral ACC brain regions of 8‐week‐old SST‐Cre mice. On the 28th day of virus expression, the bilateral whiskers of the mice were removed, and behavioral testing began on the next day. The specificity of the virus was tested (Figure 7B). The three‐chamber test results showed that chemogenetic inhibition of ACC SST neurons improved social novelty disorders in adult BWT mice, without affecting their social preference stage (Figure 7C–F).

Subsequently, the above results were further validated through optogenetic methods. The flowchart of the optogenetic experiment design is given in Figure 7G. The rAAV‐EF1a‐DIO‐mCherry or rAAV‐EF1a‐DIO‐eNpHR‐mCherry virus was injected into the bilateral ACC of 8‐week‐old SST‐Cre mice. On the 28th day of virus expression, the bilateral whiskers of the mice were removed, and behavioral testing began on the next day. The specificity of the virus was tested (Figure 7H).

The three‐chamber test results showed that optogenetic inhibition of ACC SST neurons improved social novelty disorders in adult BWT mice, without affecting their social preference stage (Figure 7I–L).

## 4 DISCUSSION

Previous studies have found that adverse experiences during childhood can lead to abnormal behaviors in adulthood, but the specific mechanisms are still unclear. Our study reveals for the first time that chronic pain experiences and tactile abnormalities during childhood lead to different social dysfunction behaviors and regulatory mechanisms in adulthood. The PV neurons of the ACC mainly regulate chronic pain in childhood to induce social preference behavior disorders in adults, while the SST neurons of the ACC mainly regulate tactile abnormalities in childhood to induce social novelty behavior disorders in adults.

Inflammatory and neuropathic pain do not affect social preference disorders in mice but can lead to social novelty stage disorders. When inflammatory pain returns to normal, the social novelty disorders caused by pain also normalize.^12^ Another study found that the social ability and social novelty preferences of the neuropathic pain model (SNI) mice showed impairments. After drug treatment, the mechanical pain in SNI mice was significantly relieved, and the social disorders associated with pain were completely restored to normal.^22^ However, the mechanism by which pain induces social disorders is still unclear. There have been numerous studies reporting the involvement of the ACC in pain regulation,^19^ among which the PV and SST neurons of the ACC play different functions. The main inhibitory effect on pain is exerted by PV interneurons, rather than SST interneurons.^23^ PV neurons target the cell body and dendrites of excitatory neurons, exerting inhibitory effects on pyramidal neurons, while SST neurons target the dendrites of pyramidal neurons, promoting arousal.^24^ Previous reports have also shown that PV neurons in the ACC can regulate social behavior.^20^ Our results similarly show that childhood inflammatory pain leads to social preference disorders in adult mice, with an increase in the number of PV neurons in the ACC. Inhibiting the PV neurons in the ACC can improve social preference disorders in young mice injected with CFA in the soles of the feet.

The mice beard is a precise tactile terminal and an important tool for mice to perceive the world.^25^ Previous studies have attempted to remove the beard at different time periods, such as 9–16 days, 12–16 days, and 16–20 days after birth. The results showed that only removing the beard before 16 days after birth had an impact on adult social behavior.^10^ There may be a critical time window for the development of neural circuits related to touch and social behavior in mice. Mice with social impairments after BWT in childhood have more neurons activated in the CA3 region of the hippocampus during social activity.^10^ In addition to the CA3, we found that the number of SST neurons in the ACC of young beard‐pruned mice increased in adulthood, and inhibiting the SST neurons in the ACC could improve social novelty disorders induced by BWT in childhood. Previous reports have also shown that SST neurons in the ACC can regulate social behavior.^21^

Our study found that not only does BWT in childhood lead to social novelty disorders in adult mice, but BWT in adults can also cause social novelty disorders. Inhibiting the SST neurons of the ACC can improve social disorders caused by BWT in childhood, as well as social novelty disorders caused by BWT in adults.

This study had certain limitations. First, it has been increasingly recognized that sexual dimorphism can affect many aspects, such as healthy neurovascular functions,^26^ pathological brain lipid metabolism,^27^ and neurovascular disorders.^28^ There are also many studies on sexual dimorphism in social disorders related to autism.^29^, ^30^ In this study, we only observed the social preference and social novelty disorders in male mice, without further exploring whether female mice also exhibited the same behavior. Further research is needed on the mechanisms of sexual dimorphism in social disorders in ACC. In addition, this study only regulated the effects of different ACC neurons on behavior through optogenetic and chemogenetic inhibition methods, without further exploring the effects of activating corresponding neurons on behavior. More experiments are warranted in the future to verify this.

In summary, our study reports for the first time that the PV neurons of the ACC mainly regulate social preference behavior disorders in adults induced by chronic pain in childhood, while the SST neurons of the ACC mainly regulate the social novelty behavior disorders in adults induced by BWT in childhood or adulthood.

## AUTHOR CONTRIBUTIONS

Xiang‐Dong WAN, Ding‐Ding YANG performed experiments, analyzed data, and prepared the manuscript. Zi‐Qian YAN performed experiments and drafted methods section. Xue‐Qing Liu, Yi‐Fan LU and Lei‐Ting AN analyzed data, and prepared the figures. Guo‐Hong CAI conceived the project and drafted the manuscript. Sheng‐Xi WU and Fan ZHANG designed experiments, supervised the experiments, and finalized the manuscript. All the authors have read and approved the paper.

## CONFLICT OF INTEREST STATEMENT

No conflicts of interest, financial or otherwise, are declared by the authors.

## ETHICAL APPROVAL AND CONSENT TO PARTICIPATE

All animal procedures were approved by our institute animal care and use committee.

## Supporting information


Table S1.

## Data Availability

The data that support the findings of this study are available from the corresponding author upon reasonable request.
